# Zero to Hero? Reducing the Rate of Acute Kidney Injury in Transcatheter Aortic Valve Replacement: The Low Contrast Approach

**DOI:** 10.31486/toj.23.0103

**Published:** 2023

**Authors:** Manuel Giraldo-Grueso, Ryan S. Bedi, Jose Tafur-Soto, Jasmine Su, Stephen M. Spindel

**Affiliations:** ^1^ Division of Cardiothoracic Surgery, Ochsner Clinic Foundation, New Orleans, LA; ^2^ Department of Cardiology, Ochsner Clinic Foundation, New Orleans, LA; ^3^ University of Massachusetts, Amherst, MA; ^4^ The University of Queensland Medical School, Ochsner Clinical School, New Orleans, LA

**Keywords:** *Acute kidney injury*, *aortic valve disease*, *contrast media*, *transcatheter aortic valve replacement*

## Abstract

**Background:** Acute kidney injury (AKI) after transcatheter aortic valve replacement (TAVR) increases hospital stay, morbidity, and mortality, and the amount of contrast used during the procedure has been linked to the occurrence of AKI. Reducing the amount of contrast used during TAVR is hypothesized to decrease AKI without compromising outcomes.

**Methods:** We conducted a single-institution retrospective analysis of patients who underwent TAVR from 2017 to 2019. Patients receiving ≤20 mL of contrast were labeled as group I, and patients receiving >20 mL of contrast were labeled as group II. Primary endpoints were 30-day mortality, AKI, and early aortic regurgitation.

**Results:** A total of 594 patients met the inclusion and exclusion criteria, with 429 patients (72.2%) included in group I and 165 patients (27.8%) included in group II. Two hundred eighteen patients (50.8%) from group I and 41 patients (24.8%) from group II had preoperative chronic kidney disease stage III or IV. The mean contrast volume was 8.5 ± 6 mL for group I and 33 ± 16 mL for group II (*P*<0.001). In group I, 13 patients (3.0%) developed AKI, and 6 (1.4%) required hemodialysis. In group II, 9 (5.5%) patients developed AKI, and 1 (0.6%) required hemodialysis. The differences between the 2 groups for AKI and hemodialysis were not statistically significant. Overall, 579 patients (97.5%) had less than moderate aortic regurgitation in the postoperative echocardiogram.

**Conclusion:** Low contrast TAVR is safe and effective and can reduce the incidence of AKI when compared to the standard contrast dose without affecting outcomes such as death and aortic regurgitation.

## INTRODUCTION

Aortic stenosis is the most common acquired valvular heart disease in North America, the prevalence increases as the population ages, and symptomatic patients have mortality rates up to 50% in 1 year.^1^ When transcatheter aortic valve replacement (TAVR) was introduced in 2002, it was intended to be used only for high-risk surgical candidates (Society of Thoracic Surgeons score >10). However, TAVR is becoming the first-line treatment for aortic stenosis since the Placement of Aortic Transcatheter Valves (PARTNER) 2 and 3 trials.^2,3^

Transfemoral TAVR has replaced surgical aortic valve replacement (SAVR) as the default treatment for most patients >80 years, and clinical, anatomic, and procedural factors guide the choice between TAVR and SAVR.^4^ Leon et al showed that TAVR had a lower composite of death from any cause and lower incidences of stroke and cardiac rehospitalization compared to SAVR during the first 2 years.^5^ However, data on long-term TAVR durability are lacking, and acute kidney injury (AKI) has been a concern since the TAVR procedure began being used because of the use of contrast.^6,7^ Contrast media can cause AKI because of its high osmotic load and because it decreases the activity of nitric oxide synthase and is cytotoxic to epithelial cells.^8^ AKI increases hospital stay, mortality, and morbidity.^6^ The amount of contrast used in a TAVR procedure has been linked to the development of AKI.^7^

We conducted this study to examine the outcomes of performing TAVR with ≤20 mL and >20 mL of contrast. We hypothesized that restricting the use of contrast during TAVR could decrease the rate of AKI without compromising short-term results.

## METHODS

### Study Design

After institutional review board approval (2021.313, approved on 2/16/2022), we conducted a single-institution retrospective analysis of patients who underwent TAVR from 2017 to 2019. Patients receiving ≤20 mL of contrast were labeled as group I, and patients receiving >20 mL of contrast were labeled as group II. We excluded patients with a left ventricular assist device or end-stage renal disease. AKI was defined according to the Valve Academic Research Consortium 3 as an increase in serum creatinine >150% within 7 days compared to baseline or an increase of >0.3 mg/dL within 48 hours of the index procedure.^9^ We retrospectively collected clinical and laboratory data for all patients at admission, immediately post-TAVR, and before hospital discharge. Primary endpoints for both groups were 30-day mortality, postoperative AKI, and early aortic regurgitation.

### Procedure and Use of Contrast

The workup for every patient included carotid artery ultrasonography; transthoracic echocardiography; coronary angiography; computed tomography scan of the heart, aorta, and peripheral vasculature; and pulmonary function testing. All TAVR procedures were done in a hybrid suite by a multidisciplinary team that included an interventional cardiologist and a thoracic surgeon. Valve options were the CoreValve (Medtronic) and SAPIEN (Edwards Lifesciences Corporation) bioprostheses; valves were chosen based on the anatomic findings on preoperative imaging.

At our institution, the goal was to limit the amount of ionized contrast used during each case to reduce the incidence of AKI. As previously stated, the risk of developing contrast-induced nephropathy is related to the dose of contrast administered.^7,10^ Cigarroa et al concluded that the threshold dose of contrast to prevent contrast-induced nephropathy should be calculated with the following equation: 5 mL contrast × body weight (in kg)/serum creatinine (in mg/dL).^11^

However, as a team, we decided to use a cutoff value of 20 mL for this analysis after determining that 20 mL was enough to obtain proper visualization and deploy the valve safely. To maximize visualization of the aortic annulus and improve TAVR positioning without the need for excessive contrast medium, 2 guide wires were routinely placed in the aortic root: 1 in the left coronary cusp and 1 in the noncoronary cusp (Figure).

**Figure.**
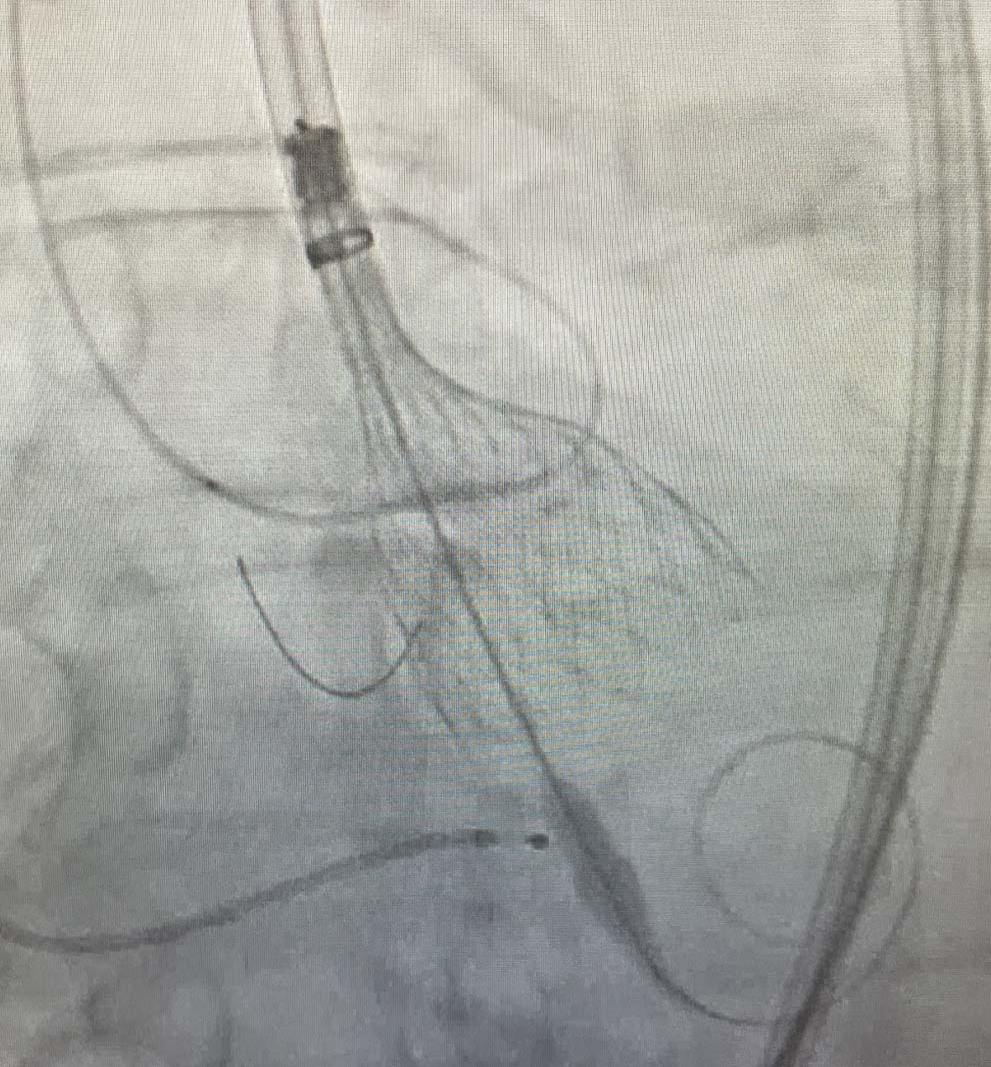
During transcatheter aortic valve replacement, 2 guide wires were placed in the aortic root: 1 in the left coronary cusp and 1 in the noncoronary cusp.

### Statistical Methods

Baseline demographics and clinical characteristics are summarized using descriptive statistics. Continuous data are presented as mean ± SD. Categorical variables are presented as frequencies and percentages. The differences between groups were ascertained using the chi-square test, Fisher exact test, and the Mann-Whitney *U* test. Known risk factors for AKI—age, diabetes mellitus, ejection fraction, and peripheral artery disease—were used in a logistic regression analysis. Significance was defined as *P*≤0.05. Stata/MP version 13.0 (StataCorp LLC) software was used for statistical calculations.

## RESULTS

Between 2017 and 2019, 597 patients underwent TAVR, and 594 met the inclusion and exclusion criteria. Four hundred twenty-nine patients (72.2%) were included in group I and 165 patients (27.8%) were included in group II (Table 1). The mean age in group I was 78 ± 9.8 years vs 77 ± 8.0 years in group II (*P*=0.121). In group I, 392 patients (91.4%) had hypertension, 93 (21.7%) had chronic obstructive pulmonary disease, and 160 (37.3%) had a prior percutaneous coronary intervention. Group II had a higher percentage of patients with diabetes mellitus than group I (49.7% vs 35.2%, respectively; *P*=0.001); however, no statistical differences were seen between groups in other demographic variables such as baseline creatinine, stroke, and ejection fraction.

**Table 1.** Demographic Variables of the Study Population, n=594

Variable	Group I, ≤20 mL, n=429	Group II, >20 mL, n=165	*P* Value
Age, years, mean ± SD	78 ± 9.8	77 ± 8.0	0.121
Diabetes mellitus	151 (35.2)	82 (49.7)	0.001
Peripheral artery disease	57 (13.3)	32 (19.4)	0.658
Hypertension	392 (91.4)	157 (95.2)	0.075
Chronic obstructive pulmonary disease	93 (21.7)	33 (20.0)	0.766
Creatinine, mg/dL, mean ± SD	1.24 ± 0.4	1.13 ± 0.3	0.071
Stroke	69 (16.1)	22 (13.3)	0.411
Ejection fraction, %, mean ± SD	54 ± 12	53 ± 11	0.252
Prior percutaneous coronary intervention	160 (37.3)	55 (33.3)	0.368
Previous cardiac operation			
Coronary artery bypass graft	102 (23.8)	45 (27.3)	0.366
Surgical aortic valve replacement	42 (9.8)	5 (3.0)	0.008
Transcatheter aortic valve replacement	2 (0.5)	0	0.381
Stage of chronic kidney disease			<0.001
I, GFR >90 mL/min/1.73 m^2^	176 (41.0)	74 (44.8)	
II, GFR 60-89 mL/min/1.73 m^2^	35 (8.2)	50 (30.3)	
III, GFR 30-59 mL/min/1.73 m^2^	170 (39.6)	40 (24.2)	
IV, GFR 15-29 mL/min/1.73 m^2^	48 (11.2)	1 (0.6)	

Note: Data are presented as n (%) unless otherwise indicated.

GFR, glomerular filtration rate.

Two hundred eighteen patients (50.8%) from group I and 41 (24.8%) patients from group II had chronic kidney disease stage III or IV (*P*<0.001). The most common vascular access was transfemoral for both groups (Table 2). No difference was seen in the type of valve used; 273 patients (45.9%) received a SAPIEN bioprosthesis, and 321 patients (54.0%) received a CoreValve bioprosthesis. The mean contrast volume was 8.5 ± 6 mL for group I and 33 ± 16 mL for group II (*P*<0.001). Forty-two patients (9.8%) from group I had zero contrast administered.

**Table 2.** Perioperative Variables

Variable	Group I, ≤20 mL, n=429	Group II, >20 mL, n=165	*P* Value
Valve in valve	44 (10.3)	5 (3.0)	0.056
Contrast, mL, mean ± SD	8.5 ± 6	33 ± 16	<0.001
Zero contrast	42 (9.8)	0	
Access			0.361
Transfemoral	398 (92.8)	150 (90.9)	
Subclavian	20 (4.7)	8 (4.8)	
Apical	11 (2.6)	7 (4.2)	

Note: Data are presented as n (%) unless otherwise indicated.

Eleven patients died during the first 30 days: 9 (2.1%) from group I and 2 (1.2%) from group II (*P*=0.477) (Table 3). Thirteen patients (3.0%) from group I developed AKI, with 6 (1.4%) requiring hemodialysis. In group II, 9 (5.5%) patients developed AKI, with 1 (0.6%) requiring hemodialysis. The differences between groups for AKI and hemodialysis were not significant, and no significant differences were seen in the postoperative variables of stroke, bleeding requiring surgery, permanent pacemaker implantation, or aortic regurgitation. Overall, 579 patients (97.5%) had less than moderate aortic regurgitation in the postoperative echocardiogram. The logistic regression analysis results for AKI highlighted diabetes mellitus as a risk factor (Table 4).

**Table 3.** Postoperative Variables

Variable	Group I, ≤20 mL, n=429	Group II, >20 mL, n=165	*P* Value
Death within 30 days	9 (2.1)	2 (1.2)	0.477
Stroke	3 (0.7)	4 (2.4)	0.080
Conversion to open surgery	0	0	
Bleeding requiring surgery	1 (0.2)	0	0.535
Acute kidney injury	13 (3.0)	9 (5.5)	0.146
Need for hemodialysis	6 (1.4)	1 (0.6)	0.435
Permanent pacemaker implantation	75 (17.5)	25 (15.1)	0.744
Aortic regurgitation			0.211
None/trace	328 (76.5)	136 (82.4)	
Mild	88 (20.5)	27 (16.4)	
Moderate	13 (3.0)	2 (1.2)	
Severe	0	0	

Note: Data are presented as n (%).

**Table 4.** Logistic Regression Analysis for Acute Kidney Injury

Predictor	*B*	95% CI for *B*	SE *B*	Significance
Age	–0.48	0.879, 1.033	0.41	0.243
Diabetes mellitus	1.9	0.034, 0.589	0.729	0.007
Preoperative ejection fraction	–0.055	0.91, 0.985	0.02	0.007
Peripheral artery disease	–1.4	0.062, 0.826	0.66	0.024

*B*, unstandardized regression weight; SE *B*, standard error for *B*.

## DISCUSSION

AKI is a prevalent complication of TAVR.^12^ Studies have shown that the 30-day mortality rate for patients with AKI following TAVR ranges from 10% to 30% compared to 2% to 15% for those without AKI, and the prevalence of AKI requiring hemodialysis after TAVR varies between 0% and 21%.^13^ The mechanism of AKI following TAVR is multifactorial; however, many believe the amount of contrast plays a significant role.^7,10,14^

Patients undergoing TAVR are generally older than patients undergoing SAVR and usually have more comorbidities.^15^ Patients who develop AKI after TAVR have higher in-hospital mortality, length of stay, and costs compared to patients without AKI. Preoperative risk factors for developing AKI after TAVR include chronic kidney disease, congestive heart failure, history of stroke, peripheral vascular disease, and use of contrast.^16^

Although the mechanism for contrast-induced nephropathy is not entirely understood, studies have shown that contrast creates an environment of inflammation, free radicals, and oxidative stress in the renal medulla, leading to programmed cell death.^17^ Hydrating with normal saline or sodium bicarbonate and limiting the amount of contrast can prevent injury.^10^

We limit the amount of contrast used in all our patients to decrease the chance of postoperative AKI, and our findings show that using ≤20 mL of contrast was safe and effective in our population. Our patients were older and had a higher rate of diabetes and hypertension compared to the patients in the Helgason et al study.^18^ Additionally, half of the patients from group I had stage III or IV kidney disease. Yet our rates of AKI (3% in group I and 5.5% in group II) were lower than the rate of 22.5% reported in the Helgason et al study,^18^ and the rates reported in the literature are 3.4% to 57%.^6,19^ Performing a low contrast TAVR could be a safe and effective way to reduce postoperative kidney dysfunction.

Some difference in the rate of AKI was seen between the 2 groups, but the difference was not statistically significant. Worth mentioning is that half of the patients from group I had chronic kidney disease stage III or IV, and as stated earlier, chronic kidney disease is an important risk factor for the development of AKI in the postoperative period.^16^ The mean contrast volume for group II was only 33 mL; this volume is lower than common practice,^20^ which is likely the reason group II had a lower or comparable rate of postoperative AKI than the rates reported in the literature.^6,19^

No consensus has been reached on AKI prevention. Introducing the KDIGO bundle^21^ can decrease the rate of postoperative AKI. The KDIGO bundle involves the discontinuation of nephrotoxic agents and angiotensin-converting enzyme inhibitors, close hemodynamic and creatinine monitoring, and avoidance of hyperglycemia. We use this bundle at our institution, and it probably helped decrease the rate of AKI. The EchoNavigator (Philips), fluoroscopy fusion images, and echocardiography are tools that help reduce the amount of contrast.^22^ Kornowski and Tang proposed a multiprong strategy to reduce the risk of postoperative AKI that includes avoiding nephrotoxic agents, reducing contrast exposure during preoperative workup with computed tomographic angiography and during the TAVR procedure, avoiding prolonged hemodynamic changes, and consulting nephrology for patients with chronic kidney disease.^14^

Despite the use of low doses of contrast, we had rates of postoperative aortic regurgitation similar to those published in the literature. The European Sentinel Registry of Transcatheter Aortic Valve Implantation showed that grade 2 aortic regurgitation was present in 7.7% of the patients and grade 3 was present in 1.3% in the predischarge echocardiography study.^19^ Grade 2 (moderate) aortic regurgitation was present at discharge in 2.5% of our patients (15/594).

Limitations of this study are that it is a single institution retrospective analysis without a control group. The procedures were done by an experienced team that is accustomed to performing low contrast TAVR, and long-term results were not analyzed.

## CONCLUSION

Low contrast TAVR was a safe and effective approach in our patient population, successfully reducing the incidence of AKI when compared to a standard contrast dose, while maintaining positive outcomes in terms of mortality, aortic regurgitation, and the necessity for a permanent pacemaker.

## Notes

*This article meets the Accreditation Council for Graduate Medical Education and the American Board of Medical Specialties Maintenance of Certification competencies for Patient Care, Medical Knowledge, and Practice-Based Learning and Improvement.*
